# Contribution of Lipid Oxidation and Ferroptosis to Radiotherapy Efficacy

**DOI:** 10.3390/ijms222212603

**Published:** 2021-11-22

**Authors:** Ashley N. Pearson, Joseph Carmicheal, Long Jiang, Yu Leo Lei, Michael D. Green

**Affiliations:** 1 Program in Biomedical Sciences, University of Michigan Medical School, Ann Arbor, MI 48109, USA; anpears@med.umich.edu; 2 College of Medicine, University of Nebraska Medical Center, Omaha, NE 68198, USA; joseph.carmicheal@unmc.edu; 3 Department of Radiation Oncology, University of Michigan School of Medicine, Ann Arbor, MI 48109, USA; lojiang@med.umich.edu; 4 Department of Periodontics and Oral Medicine, University of Michigan, Ann Arbor, MI 48109, USA; leiyuleo@med.umich.edu; 5 Department of Otolaryngology, University of Michigan, Ann Arbor, MI 48109, USA; 6 Rogel Cancer Center, University of Michigan, Ann Arbor, MI 48109, USA; 7 Department of Microbiology and Immunology, University of Michigan School of Medicine, Ann Arbor, MI 48109, USA; 8 Graduate Program in Immunology, University of Michigan School of Medicine, Ann Arbor, MI 48109, USA; 9 Veterans Affairs Ann Arbor Healthcare System, Ann Arbor, MI 48105, USA

**Keywords:** lipid oxidation, ferroptosis, radiotherapy

## Abstract

Radiotherapy promotes tumor cell death and senescence through the induction of oxidative damage. Recent work has highlighted the importance of lipid peroxidation for radiotherapy efficacy. Excessive lipid peroxidation can promote ferroptosis, a regulated form of cell death. In this review, we address the evidence supporting a role of ferroptosis in response to radiotherapy and discuss the molecular regulators that underlie this interaction. Finally, we postulate on the clinical implications for the intersection of ferroptosis and radiotherapy.

## 1. Introduction

Radiotherapy is used in the curative and palliative management of more than half of all cancer patients [1]. Unfortunately, resistance to radiotherapy limits its therapeutic efficacy. Radiotherapy induces multiple forms of regulated and unregulated cell death [2]. Without an improved understanding of the molecular mechanisms behind radiotherapy induced cell death, the design of new therapeutic strategies which augment radiotherapy efficacy is limited. Recently, it has been found that radiotherapy generated lipid oxidation and ferroptosis [3,4,5]. In this review, we will discuss the connections between lipid oxidation, ferroptosis, and radiotherapy, as well as elaborate on the clinical implications of ferroptosis modulators as radiosensitizers.

## 2. Molecular Regulators of Ferroptosis

Lipids are a critical buffer to reactive oxygen species (ROS) and exist in an equilibrium between oxidized and reduced states. This balance is known as lipid redox homeostasis, a process which is essential for ensuring cell survival [6]. One of the lethal outcomes of redox imbalance is ferroptosis, a unique form of cell death induced by iron-dependent lipid peroxidation [7]. The term “ferroptosis” was only recently described, although the phenomenon of lipid oxidative damage has been observed for decades [8]. By the 1980s, the role of lipid oxidation in cell stress was well-described [9,10]. It was not until more recently that it was discovered that excessive lipid oxidation leads to cell death. However, the precise mechanism through which lipid oxidation causes ferroptosis is still unknown [11,12]. Lipid peroxidation is the key driver of ferroptosis [13,14]. Specifically, oxidation of polyunsaturated fatty acids (PUFA), mediated by lipoxygenases and the intracellular iron pool, which is in turn regulated by phosphorylase kinase catalytic subunit gamma 2 (PHKG2), drives ferroptosis [15]. Synthesis of PUFAs containing ether phospholipids provides substrates that are then peroxidated to drive ferroptosis [16]. Acyl-coenzyme A synthetase long-chain family member 4 (ACSL4) esterifies CoA to free fatty acids, with a preference for long PUFAs [17]. ACSL4 inhibits redox homeostasis by allowing for accumulation of oxidized lipids within the plasma membrane [18]. In contrast, it has been shown that introduction of monounsaturated fatty acids inhibits ferroptosis by decreasing membrane ROS content via acyl-CoA synthetase long-chain family member 3 (ACSL3) activity [19]. Another aspect to consider is mitochondrial electron transport chain activity, which contributes to the generation of endogenous lipid radicals to induce ferroptosis [20]. Thus, lipid metabolism and biosynthesis regulate ferroptosis.

Lipid oxidation and ferroptosis are limited by multiple pathways. One critical regulator is glutathione peroxidase 4 (GPX4), a mammalian glutathione peroxidase which inhibits lipid peroxidation and contributes to redox homeostasis by catalyzing the reduction of lipid peroxides [21,22,23]. In the context of B cell lymphomas and renal cell carcinomas, GPX4 regulates induction of ferroptosis. RSL-3, a pharmacological ferroptosis inducer, drives colorectal cancer cell death via direct binding and inactivation of GPX4, resulting in loss of redox homeostasis [22]. Other defenses include ferroptosis suppressor protein 1 (FSP1), which functions as a coenzyme Q10 oxidoreductase and restores the antioxidant pool to suppress ferroptosis [24]. These data highlight that multiple enzymatic pathways act in concert to limit lipid peroxidation induced cell death. Antioxidants are required for the enzymatic reduction of lipids and prevention of ferroptosis. Tetrahydrobiopterin, generated by GTP cyclohydrolase I, is a hydroxylase cofactor and antioxidant which limits ferroptosis [25,26]. Cysteine, the reduced form of cystine, is the rate-limiting precursor for the antioxidant glutathione (GSH). In the 1950s, Eagle demonstrated the importance of cystine presence for Hela cell survival [27]. Depletion of GSH has been linked to diminished GPX4 activity and ferroptosis in cancer cells [28,29]. Cystine import is regulated by system x_c_^−^, a cystine-glutamine anti-transporter [11] composed of a heavy chain (Solute Light Chain, SLC3A2) and a light chain (SLC7A11) [30]. Interestingly, tumor protein 53 (p53) increases expression of SLC7A11, driving cystine import to restrain oxidative stress and thus prevent ferroptotic cell death in cancer cells [31]. In murine pancreatic cancer cells, deletion of SLC7A11 was sufficient to decrease cystine import, downregulate GSH activity, and induce tumor ferroptosis [28]. Additionally, interferon-γ from CD8^+^ T cells can promote tumor cell ferroptosis by downregulating SLC3A2 and SLC7A11, impairing tumor cystine uptake and disrupting tumor cell redox homeostasis [32]. Other regulators of system x_c_^−^ include nuclear factor erythroid factor 2-related factor 2 and kelch-like ECH-associated protein 1 (NRF2-KEAP1) signaling [33]. This may be exploited to treat cancer by sensitizing it to radiotherapy [5]. Further evidence of the importance of antioxidants to ferroptosis comes from metabolic perturbations. Use of a glutamine antagonist increased T cell-mediated antitumor immunity in several murine cancer models [34]. However, Gao et al. showed that glutamine supplementation induced ferroptosis in mouse embryonic fibroblasts, and Rossler et al. demonstrated that elevated glutamate contributes to cell death in HT22 cells [35,36]. More work is needed to better define the role of glutamine on ferroptosis in cancer. Thus, metabolic import and antioxidant biosynthetic pathways regulate ferroptosis. 

**Figure 1:**
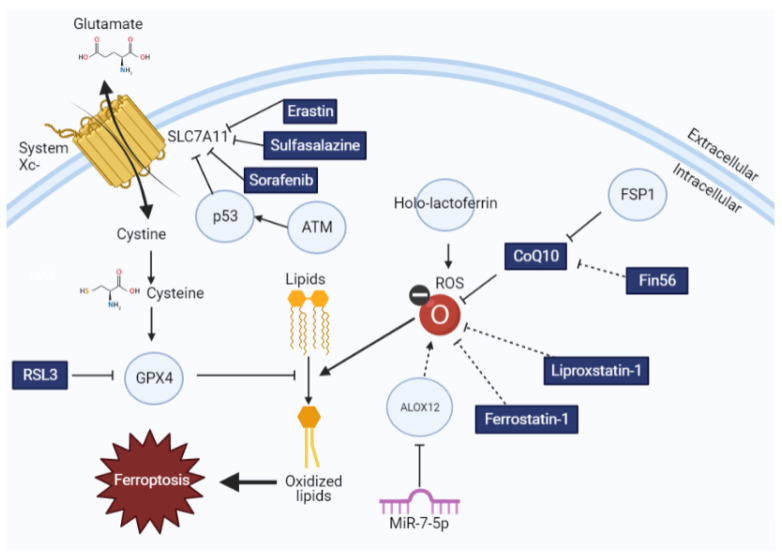
The impact of ferroptotic inducers and inhibitors on lipid redox homeostasis. Glutathione synthesis is regulated by cystine import by system x_c_^−^, which is then used by GPX4 to oppose lipid oxidation. This process can be modulated by different ferroptosis inducers and inhibitors, as explained here. Drugs and small molecules are indicated in rectangles, whereas proteins are indicated with circles. Solid arrows indicate direct interactions, whereas dotted arrows indicate correlative interactions.

Labile iron radicals can directly generate oxygen radicals via Fenton chemistry [37] and promote lipid peroxidation directly [38]. Iron can also indirectly promote ferroptosis as a cofactor in the enzymes which promote lipid oxidation [7]. For example, cytochrome P450 oxidoreductase depends on the cycling of iron between ferric and ferrous states to enable lipid peroxidation [39]. Lipoxygenase enzymes, which are iron dependent and promote lipid oxidation, also promote ferroptosis [40]. It has been shown that pharmacologic iron chelators as well as physiologic iron chelators can inhibit ferroptosis, although the precise mechanism by which this occurs is not yet known [7,41]. These data underscore the necessity of iron to induce ferroptosis. Collectively, this work highlights the multiple modules that regulate lipid redox homeostasis (Figure 1).

## 3. Evidence of Lipid Oxidation and Ferroptosis Following Radiotherapy

Recent work has demonstrated a clear connection between radiotherapy, lipid redox homeostasis, and ferroptosis. Biochemical studies show that multilamellar liposomes treated with therapeutically relevant doses of radiotherapy undergo peroxidation as well as lipid fragmentation, leading to the rupture of a model membrane [42]. In vitro studies have shown that absorption of radiotherapy by water leads to the formation of oxygen radicals, which subsequently attack PUFAs to cause lipid peroxidation [37,43]. Radiotherapy has been shown to increase lipid oxidation in a dose-dependent manner when quantified by ROS sensitive fluorescent dyes which localize to lipid membranes [5]. These studies demonstrate a clear link between radiotherapy and lipid peroxidation. 

The consequence of excessive lipid oxidation is ferroptosis. Lang et al. found that radiotherapy treatment of ID8 ovarian cancer cells increases lipid oxidative damage by activating the ataxia-telangiectasia mutated gene (ATM) to suppress SLC7A11 expression. This resulted in loss of lipid redox homeostasis and initiation of ferroptosis. Inhibition of ATM rescued the cells from radiotherapy-induced ferroptosis [5]. Lei et al. showed that radiotherapy treatment of different non-small cell lung cancer (NSCLC) lines induced ROS production, lipid peroxidation, and increased ACSL4 expression. Deletion of ACSL4 inhibited ferroptosis by reducing lipid peroxidation [3]. Ye et al. provided functional evidence that administration of ferroptosis inducers improved the cell-killing effects of radiotherapy, both in fibrosarcoma cells in vitro and human patient murine xenografts of adenocarcinoma and glioma [4]. Shrunken mitochondria are a hallmark of ferroptosis, and Lei et al. demonstrated that cancer cells treated with ionizing radiotherapy were characterized by shrunken mitochondria, further suggesting a link between radiotherapy and ferroptosis induction [3]. Finally, increases in tumoral lipid oxidation following radiotherapy based neoadjuvant treatment in patients with esophageal cancer is associated with improved locoregional control and OS [3]. Taken together, these data suggest that radiotherapy promotes lipid oxidation and ferroptosis.

## 4. Therapeutic Opportunities to Enhance Radiotherapy Efficacy via Ferroptosis Induction

Ferroptotic sensitivity is dictated by the proportion of PUFA in the lipid membrane [44]. Interestingly, genetic perturbation of lipid composition modulates radiotherapy sensitivity. ACSL4 knockout, which results in diminished PUFA lipid synthesis, abolishes radiotherapy efficacy in vitro and in vivo [5]. Conversely, ACSL3 knockout, which limits monounsaturated fatty acid lipid synthesis in the cell membrane, augments radiotherapy efficacy [5]. Additionally, lipid metabolism regulates lipid membrane composition. Repeated radiotherapy exposure to fractionated radiotherapy can generate cancer cell lines resistant to radiation [45]. Work has shown that in cervical cancer cells, upregulation of MiR-7-5p promotes radiotherapy resistance by silencing arachidonate 12-lipoxygenase (ALOX12) and other components of ferroptosis signaling, thus limiting radiotherapy efficacy [45]. Together, these data suggest that lipid metabolism can be therapeutically targeted to improve radiotherapy efficacy.

Cystine import is critical for glutathione biosynthesis and maintenance of the antioxidant pool within the cell. Upregulation of SLC family members that regulate cystine import has been tied to acquired radiotherapy resistance in vitro [46]. Recombinant enzymes that degrade cysteine and cystine promote radiotherapy sensitivity of melanoma and ovarian tumors in vivo [5]. Further, pharmacologic inhibitors of SLC7A11, the critical antiporter responsible for cystine uptake, increase radiotherapy sensitivity [4]. Erastin, an SLC7A11 inhibitor, sensitizes radiotherapy resistant NSCLC cancer lines [47]. Another SLC7A11 inhibitor, sulfasalazine, has been shown to promote radiotherapy efficacy in cell line xenograft and patient derived xenograft murine models of lung cancer. Interestingly, sulfasalazine alone did not impact the size of tumors in the absence of radiation in this study [3]. Further, sorafenib, a tyrosine kinase inhibitor that inhibits SLC7A11, sensitizes fibrosarcoma and lung adenocarcinoma xenografts to radiotherapy by increasing lipid peroxidation without increasing the DNA damage profile compared to radiation alone [4]. Radiation efficacy was shown to synergize with direct GPX4 inhibition via RSL-3 to diminish clonogenic survival in multiple models when compared to radiation or RSL-3 alone. This work provides functional evidence that administration of ferroptosis inducers improves radiotherapy efficacy in different contexts, including fibrosarcoma cells in vitro, murine xenografts of lung adenocarcinoma and fibrosarcoma in vivo, and glioma patient derived slice cultures ex vivo [4].

Corroborating this preclinical data, high NRF2 and SLC7A11 expression has been associated with diminished radiotherapy induced ferroptosis and decreased lipid oxidation, as well as radiotherapy resistance in patients with esophageal cancer [48]. In head and neck cancer, treatment with artesunate to inhibit NRF2 increased ferroptosis in cancer cells [49]. KRAS, an oncogene mutated in approximately 25% of human cancers, regulates NRF2 signaling [50]. In pancreatic cancer, KRAS signaling upregulating NRF2 led to chemoresistance via increases in glutaminolysis [51]. KRAS mutant lung cancer cells showed resistance to erastin-induced ferroptosis [52]. This suggests that KRAS can promote cancer resistance to ferroptosis by signaling through NRF2 to upregulate system x_c_^−^. Together, these data highlight that cystine transporters are a novel and targetable mechanism to augment radiotherapy efficacy.

Iron metabolism and oxidation have been tied not only to ferroptosis but also radiotherapy efficacy. Holo-Lactoferrin is a radiosensitizer which increases total iron content, promotes ROS, and facilitates lipid oxidation to enhance radiotherapy efficacy through ferroptosis [53]. Depletion of mitochondrial antioxidants including Coenzyme Q with FIN56, a known ferroptosis inducer, diminishes cancer cell survival following radiotherapy. The glycoprotein collectrin improves radiotherapy sensitivity through ferroptosis induction in the setting of hepatocellular carcinoma [54]. 

## 5. Therapeutic Opportunities to Limit Radiotherapy Toxicity via Ferroptosis Inhibition

Normal tissue toxicity following radiotherapy can cause significant morbidity in cancer patients. Technological advances in radiotherapy planning and delivery have led to striking decreases in patient adverse events. However, for patients in which large treatment fields or ablative doses are required for tumor control, radiotherapy can produce significant normal tissue toxicity in adjacent, healthy organs. Focal administration of therapeutic doses of radiotherapy can induce a hyperinflammatory cytokine response via the release of cardiolipin and phosphatidylserine lipid oxidation in the lung [55] as well as malondialdehyde lipid oxidation in the liver [56]. In preclinical models receiving thoracic irradiation, ferroptosis inhibitors limit cytokine release following radiotherapy, thereby decreasing inflammation and reducing lung fibrosis [57,58]. Thus, radiotherapy-induced ferroptosis may contribute to late effects following radiotherapy in slow-growing tissues. 

Exposure to whole body radiation in the setting of extraterrestrial travel or incidental radiation exposure can be potentially lethal. Multiorgan dysfunction contributes to morbidity and mortality in this setting, including gastrointestinal and hematopoietic injuries [59]. Additionally, multiple cancers required low dose total body irradiation (TBI) as adjuvant or ablative treatment, and this also has associated toxicities [60]. It has long been understood that organs with rapidly dividing cells, including the intestinal epithelium and hematopoietic precursors, respond acutely to radiotherapy. It has been suggested that total body irradiation increases DNA damage and antioxidant responses to induce normal tissue toxicity [61]. Interestingly, TBI has been shown to increase bone marrow lipid oxidation and decrease bone marrow Vitamin E, a lipophilic antioxidant, in a dose-dependent manner [62]. This suggests that ferroptosis may contribute to total body irradiation toxicity and may also contribute to acute effects following radiotherapy treatment in rapidly dividing tissues. 

Consistent with this, polycysteine derivatives, which increase the antioxidant pool and promote GPX4 activity, limit lethal whole body radiotherapy toxicity in preclinical models. Further, treatment with polycysteine derivatives limited gastrointestinal and hematopoietic toxicity as well as radiotherapy induced lung disease in murine models. Mechanistic studies showed that polycysteine diminished lipid oxidation and restored the GSH pool following radiotherapy [63]. Yet another molecule, arachidonate-15-lipoxygenase-1 (ALOX15), promotes lipid oxidation and contributes to the induction of ferroptosis [64]. Baicalein, an ALOX15 inhibitor, has been shown to normalize inflammatory cytokines induced by total body irradiation and improves the survival of mice treated with TBI even when administered post radiation exposure [65]. Collectively, these data suggest that ferroptosis contributes to normal tissue toxicity after radiation exposure and suggests that manipulation of ferroptotic induction may provide radioprotection for healthy tissues. At present, lipophilic antioxidant ferroptosis inhibitors include liproxstatin-1 and ferrostatin-1, which are currently unsuitable for in vivo administration, although medicinal chemistry approaches are improving the pharmacodynamics of these compounds [66]. 

## 6. Conclusions and Future Directions

There is now compelling literature that radiotherapy induces lipid oxidation and ferroptosis in tumors. This adds to the growing body of evidence that radiotherapy can provide tumor control by inducing programmed forms of cell death, including apoptosis [67,68], necroptosis [69], autophagy [70], and now ferroptosis. Additionally, radiotherapy can induce unregulated forms of tumor cell death including mitotic catastrophe, necrosis, and senescence [71,72]. Recent work has demonstrated that stress granules are associated with cancer chemoresistance and may provide a link between ferroptosis and radioresistance [73]. Additional work is required to understand whether radiotherapy dose, fractionation, sequencing, and source as well as cancer type and tumor microenvironment alter the relative contribution of each form of cell death following radiotherapy. 

The radiosensitization agents currently most clinically utilized include platinum compounds, alkylating agents, inhibitors of DNA synthesis, and topoisomerase inhibitors [74]. Emerging strategies for radiosensitization include targeting the DNA damage response [75]. However, these strategies converge on augmenting DNA damage. Ferroptosis inducers have been shown to sensitize preclinical cancer models to radiotherapy, suggesting that augmentation of lipid damage may offer a novel therapeutic target that may prove to be an invaluable addition to the anticancer therapeutic armamentarium. Importantly, studies suggest that ferroptosis following radiotherapy is independent of DNA damage [4]. As the DNA damage response and lipid oxidation both induce a cellular stress response with common molecular modulators including p53 and ATM [76], additional work is required to understand the nature of cross talk between DNA and lipid oxidative damage following radiotherapy. Additional studies are also required to establish the optimal ferroptotic agent, administration schedule, and dose to advance to clinical trials in combination with radiotherapy (Table 1).

Radiation damage to adjacent healthy organs and tissues can cause significant morbidity in cancer patients. This radiotherapy toxicity in normal tissue may also rely on lipid oxidation and ferroptosis. Additional studies are required to understand whether targeting ferroptosis to augment radiotherapy efficacy widens or narrows the therapeutic index. 

## Figures and Tables

**Table 1:** Current clinical trials of drugs targeting ferroptosis. Sorafenib and sulfasalazine, neither of which were originally developed for the treatment of ferroptosis, may serve as ferroptosis inhibitors due to their ability to decrease the activity of SLC7A11 [77,78].

Drug Name	Relevant Dates	Original Target	Current Number of Ongoing Clinical Trials	Ferroptotic Target
Sorafenib	2000: Entered clinical trials	MAPK Cascade in Cancer	92	SLC7A11
Sulfasalazine	1950: Approved for clinical use	Rheumatoid arthritis	13	SLC7A11
